# Interactions between Siglec-8 and endogenous sialylated cis ligands restrain cell death induction in human eosinophils and mast cells

**DOI:** 10.3389/fimmu.2023.1283370

**Published:** 2023-10-20

**Authors:** Yun Cao, Clayton H. Rische, Bruce S. Bochner, Jeremy A. O’Sullivan

**Affiliations:** ^1^ Division of Allergy and Immunology, Northwestern University Feinberg School of Medicine, Chicago, IL, United States; ^2^ Department of Biomedical Engineering, Northwestern University McCormick School of Engineering, Evanston, IL, United States

**Keywords:** Siglec-8, eosinophil, mast cell, sialic acid, cis ligand, cell death

## Abstract

Sialic acid-binding immunoglobulin-like lectin (Siglec)-8 is a sialoside-binding receptor expressed by eosinophils and mast cells that exhibits priming status- and cell type-dependent inhibitory activity. On eosinophils that have been primed with IL-5, GM-CSF, or IL-33, antibody ligation of Siglec-8 induces cell death through a pathway involving the β2 integrin-dependent generation of reactive oxygen species (ROS) via NADPH oxidase. In contrast, Siglec-8 engagement on mast cells inhibits cellular activation and mediator release but reportedly does not impact cell viability. The differences in responses between cytokine-primed and unprimed eosinophils, and between eosinophils and mast cells, to Siglec-8 ligation are not understood. We previously found that Siglec-8 binds to sialylated ligands present on the surface of the same cell (so-called cis ligands), preventing Siglec-8 ligand binding in trans. However, the functional relevance of these cis ligands has not been elucidated. We therefore explored the potential influence of cis ligands of Siglec-8 on both eosinophils and mast cells. De-sialylation using exogenous sialidase profoundly altered the consequences of Siglec-8 antibody engagement on both cell types, eliminating the need for cytokine priming of eosinophils to facilitate cell death and enabling Siglec-8–dependent mast cell death without impacting anti–Siglec-8 antibody binding. The cell death process licensed by de-sialylation resembled that characterized in IL-5–primed eosinophils, including CD11b upregulation, ROS production, and the activities of Syk, PI3K, and PLC. These results implicate cis ligands in restraining Siglec-8 function on eosinophils and mast cells and reveal a promising approach to the selective depletion of mast cells in patients with mast cell-mediated diseases.

## 1 Introduction

Eosinophils and mast cells (MCs) are innate immune effector cells that, jointly or independently, through their inappropriate expansion, accumulation, and activation, contribute to a range of diseases, including allergy, asthma, chronic spontaneous urticaria, eosinophilic granulomatosis with polyangiitis, hypereosinophilic syndromes, systemic mastocytosis, and others (1–6). There is a clinical need for disease-modifying therapies for these diseases and, given the cooperativity between eosinophils and MCs and the difficulty of blocking cellular activation or the activities of released mediators, the ability to deplete both cell types would be widely beneficial in such diseases.

Sialic acid-binding immunoglobulin-like lectin (Siglec)-8 is a glycan-binding receptor highly and selectively expressed by eosinophils and MCs (7, 8). Like many other Siglecs, Siglec-8 possesses in its cytoplasmic domain an immunoreceptor tyrosine-based inhibitory motif (ITIM) and an ITIM-like motif that are thought to be critical for downstream signaling and function. Antibody engagement of Siglec-8 on cytokine (i.e., interleukin (IL)-5, granulocyte-macrophage colony-stimulating factor (GM-CSF), or IL-33)-primed eosinophils induces cell death through a pathway involving CD11b/CD18 integrin–mediated adhesion and reactive oxygen species (ROS) production (9–12); however, in the absence of cytokine priming or extensive crosslinking, no consistent effect of Siglec-8 engagement on eosinophils has been observed (10). In contrast, antibody ligation of Siglec-8 on MCs has been described to cause functional inhibition but not cell death (13–15). The reasons underlying these discrepant outcomes of Siglec-8 engagement have thus far not been elucidated.

While antibodies are often used to target or study Siglecs, the physiological ligands of these receptors are putative glycan structures that terminate with the negatively charged sugar sialic acid. These ligands may be found on soluble structures or on the surfaces of other cells (trans ligands) or on the surface of the same cell expressing the Siglec (cis ligands). Cis ligands may hinder Siglecs from interacting with trans ligands and thereby create an affinity/avidity threshold for such interactions (“masking” the Siglec), and cis interactions are commonly discovered by observing enhanced trans ligand binding upon cleaving cell-surface sialic acid from the Siglec-expressing cell using sialidase (16–21). Cis ligands may also play a more direct functional role by sequestering the Siglec and preventing interactions with a target of inhibition. For example, CD22 (Siglec-2) on B cells is isolated away from the B cell receptor (BCR) by interactions with cis ligands, including sialic acid–dependent interactions with other molecules of CD22, which prevents CD22-mediated inhibition of BCR signaling (22–27). Alternatively, cis ligand interactions may promote inhibition by bringing inhibitory targets into close proximity with the Siglec. In mice, interactions of Siglec-G and Siglec-E with the BCR and TLR4, respectively, are dependent on sialic acid, and disruption of these interactions release Siglec-mediated inhibition of these pathways (28–30).

We have previously found that Siglec-8 interacts with sialylated cis ligands on human eosinophils and that this interaction reduces binding of a high-avidity synthetic Siglec-8 ligand in trans (31). However, the functional relevance of this interaction has not been explored. In the present study, we examine the role of sialylated cis ligands of Siglec-8 on human eosinophils and MCs and demonstrate that these ligands are responsible for restraining Siglec-8–induced cell death on both cell types. Specifically, the enzymatic removal of α2,3-linked sialic acid from the surface of these cells licenses Siglec-8 to cause cell death upon receptor ligation via a pathway that resembles that described for IL-5–licensed eosinophil death induction. This effect is dependent on the enzymatic activity of the sialidase, and no enhancement of antibody binding of Siglec-8 is observed following incubation with sialidase. These findings highlight the importance of cis ligand interactions in fundamentally altering the consequences of Siglec engagement and provide a potential pathway to deplete both eosinophils and MCs through Siglec-8.

## 2 Materials and methods

### 2.1 Human eosinophil and mast cell isolation and culture

Written informed consent for blood donation (up to 180 mL) was obtained using an institutional review board–approved protocol at the Northwestern University Feinberg School of Medicine. Eosinophils from both allergic and non-allergic donors were purified from peripheral blood using density gradient centrifugation, erythrocyte hypotonic lysis, and CD16 immunomagnetic negative selection (Miltenyi Biotec, San Diego, CA) as described (32). Purity and viability were consistently greater than 95% as determined by Siglec-8 staining and DAPI (ThermoFisher Scientific, Waltham, MA) exclusion (33). Purified eosinophils were cultured in Roswell Park Memorial Institute (RPMI) 1640 medium with 10% fetal calf serum (FCS) and antibiotics (all from ThermoFisher Scientific) as well as with or without 30 ng/mL recombinant human (rh)IL-5 (R&D Systems, Minneapolis, MN) for 18–24 hr as indicated.

Primary human MCs were isolated via enzymatic digestion from de-identified normal human skin specimens obtained through the Cooperative Human Tissue Network (CHTN) as previously described (34). Following enzymatic digestion, digested tissue was filtered through a wire mesh and collected cells were washed twice. The resulting cell pellet was resuspended in wash buffer and kept on ice. Collected cells were then processed through a Percoll (Sigma-Aldrich, St. Louis, MO) gradient. Cells were collected, washed, and plated at a concentration of 5×10^5^ cells/mL in serum-free X-VIVO medium (Lonza, Basel, Switzerland) supplemented with 100 ng/mL recombinant human stem cell factor (rhSCF) (Peprotech, Cranbury, NJ, USA). Cells were fed weekly and maintained at a concentration of 5×10^5^ cells/mL. Skin-derived MCs were used in experiments after 8-12 weeks in culture when MC purity exceeded 95%.

### 2.2 Enzymatic and pharmacologic treatments of eosinophils and MCs

Eosinophils and MCs were incubated with *V. cholerae* sialidase generously provided by Dr. Ronald Schnaar (Johns Hopkins University School of Medicine, Baltimore, MD) at the indicated activities. To determine the necessity of sialidase enzymatic activity or the activities of various signaling molecules, eosinophils were pre-incubated with pharmacologic inhibitors. In some experiments, eosinophils were pre-incubated with the sialidase inhibitor 2,3-dehydro-2-deoxy-N-acetylneuraminic acid (DANA) (Sigma-Aldrich) at a final concentration of 250 μM 30 min prior to the addition of sialidase. Pre-incubation with inhibitors of spleen tyrosine kinase (Syk) (OXSI-2, 667 nM; Cayman Chemical, Ann Arbor, MI), phosphoinositide 3-kinase (PI3K) (LY294002, 5 μM; Selleckchem, Houston, TX), or phospholipase C (PLC) (U73122, 100 nM; Tocris Biosciences, Bristol, UK) was initiated 30 min prior to antibody ligation of Siglec-8 at 37°C, with each pharmacologic agent remaining in the medium for the duration of the incubation period. Skin-derived MCs were incubated with sialidase at 300 mU/mL for 2 hrs to cleave cell-surface sialic acid prior to the addition of antibodies.

### 2.3 Assessment of CD11b upregulation, ROS production, and cell death following Siglec-8 engagement

Eosinophils (2×10^5^ cells in 200 µL medium per condition) were cultured for 18–24 h at 37°C with or without 30 ng/mL rhIL-5, then anti–Siglec-8 mAb (monoclonal antibody) (clone 2C4, generated as previously described (8)) or mouse IgG1 isotype control mAb (clone MOPC-21; Tonbo Biosciences, San Diego, CA) was added to a final concentration of 2.5 µg/mL. All treatment antibodies and controls were azide-free. Levels of cell-surface CD11b expression were assessed as previously described (11). Briefly, eosinophils were cultured for 2 h after stimulation prior to washing, staining for CD11b surface expression (clone ICRF44; BD Biosciences, San Jose, CA) in conjunction with the viability stain DAPI, and collecting data on a BD LSR II or Beckman Coulter CytoFLEX (Brea, CA) flow cytometer. To assess cell death induction as a result of Siglec-8 engagement, human eosinophils or skin-derived MCs (also at 2×10^5^ cells in 200 µL medium per condition) were incubated with antibody for 18–24 h as previously described (10, 11), and cell death was assessed by flow cytometry after fluorophore-labeled annexin V (BioLegend, San Diego, CA) and DAPI staining. MCs were additionally incubated with anti-Siglec-6 mAb (clone 767329; R&D Systems) or mouse IgG2a isotype control mAb (BioLegend) at 2.5 µg/mL. ROS levels were detected as previously described (11, 35). Briefly, eosinophils were loaded at 37°C with dihydrorhodamine 123 (DHR 123; ThermoFisher Scientific) for 15 min prior to the addition of the indicated mAb at a final concentration of 2.5 µg/mL. After 120 min, the cells were washed, stained with DAPI, and analyzed by flow cytometry. Flow cytometric data were analyzed using FlowJo software v10 (TreeStar, Ashland, OR).

### 2.4 Lectin staining

To detect cell-surface sialic acid, eosinophils (2×10^5^ cells per condition) were incubated with biotinylated *Maackia amurensis* lectin (MAL)-II or *Sambucus nigra* agglutinin (SNA) (both from Vector Laboratories, Newark, CA) at 10 μg/mL for 30 min. After washing, cell surface-bound lectin was stained with DyLight488-conjugated streptavidin (Vector Laboratories) at 10 μg/mL for 30 min and detected by flow cytometry. MAL-II and SNA signals were normalized to stained, untreated samples within each experiment. Eosinophils were stained for cell-surface sialic acid following 18-24 h of priming with 30 ng/mL IL-5 or treatment with *V. cholerae* sialidase at the indicated enzymatic activity level for 1 h. Eosinophils were washed prior to staining. Data were collected on a BD LSR II or Beckman Coulter CytoFLEX flow cytometer and analyzed using FlowJo software v10 as described above.

### 2.5 Statistical analysis

Data are presented as mean ± standard deviation unless otherwise indicated. Statistical significance was determined by one-way or two-way ANOVA and Tukey, Dunnett, or Šídák corrections for multiple comparisons as indicated using GraphPad Prism 6.0e. Statistical differences were considered significant at p < 0.05.

## 3 Results

### 3.1 Characterization of Siglec-8 cis ligands on human eosinophils

**Figure 1:**
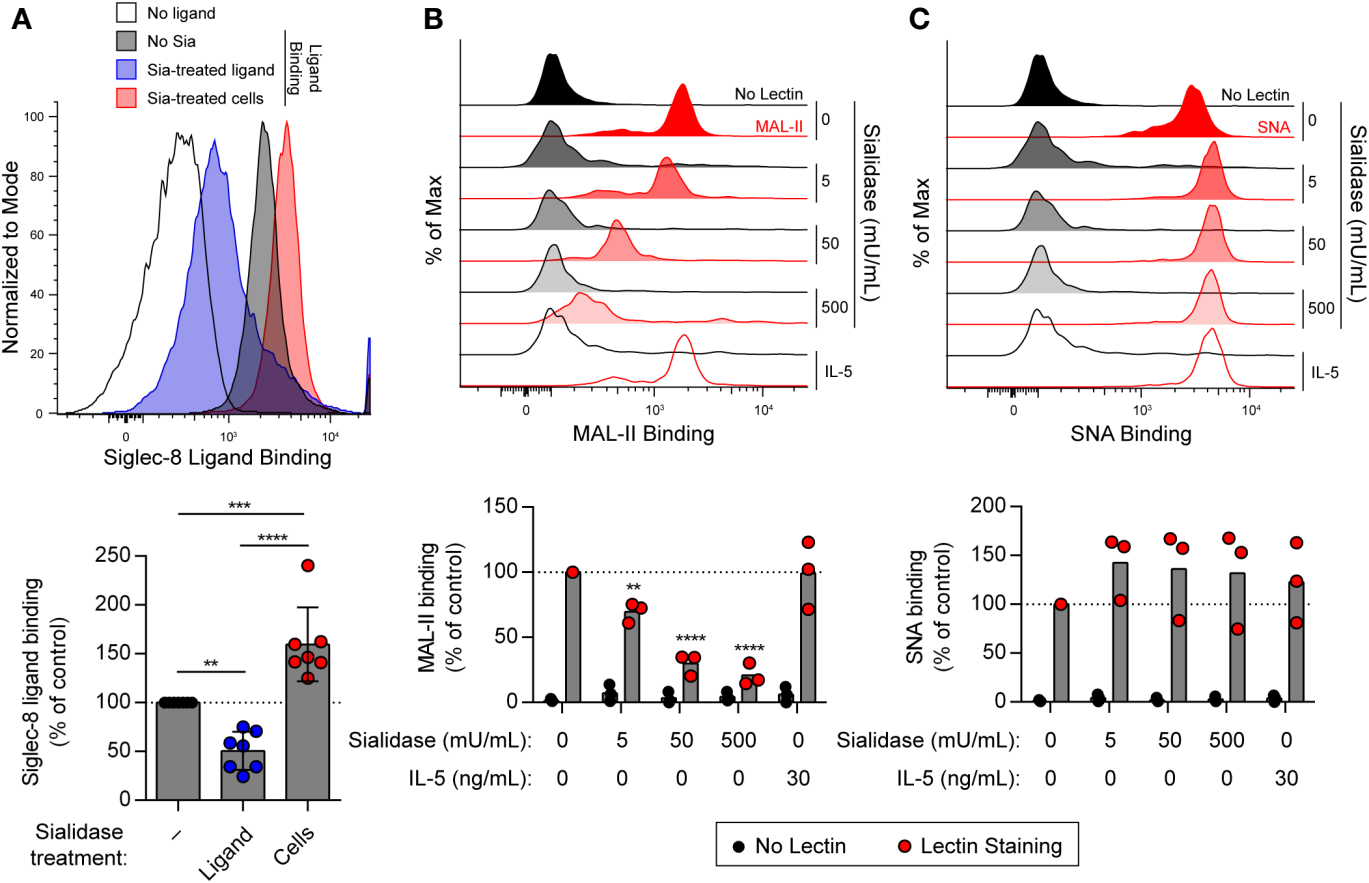
Treatment of human eosinophils with sialidase from *V. cholerae* selectively cleaves α2,3-linked sialic acids from the cell surface. **(A)** Human eosinophils isolated from peripheral blood were incubated with biotinylated 1-MDa polyacrylamide decorated with the Siglec-8 glycan ligand 6′-sulfo-3′-sialyl-LacNAc for 20 min at 4°C prior to detection of surface-bound ligand with fluorophore-conjugated streptavidin. Sialidase (Sia) from *V. cholerae* was used at 50 mU/mL for 1 hr at 37°C to cleave sialic acid from the ligand or from the eosinophils prior to ligand binding. Data are representative (top) or show the quantified normalized Siglec-8 binding to eosinophils relative to control untreated cells as well as overall mean and standard deviations of seven independent experiments (bottom). **, p<0.01; ***, p<0.001; ****, p<0.0001 by one-way ANOVA and Tukey multiple comparisons test. **(B, C)** Eosinophils were incubated with or without 30 ng/mL rhIL-5 for 18–24 hr. Unprimed cells were then treated with sialidase at the indicated activities for 1 hr prior to detection of cell-surface sialic acids by lectin binding. Surface-bound biotinylated MAL-II **(B)** or SNA **(C)** lectin was detected using fluorophore-conjugated streptavidin by flow cytometry. Similarly treated eosinophils incubated without lectin (No Lectin) were analyzed for background streptavidin binding. Data are representative (top) or show quantified normalized lectin binding from three independent experiments (bottom). **, p<0.01; ****, p<0.0001 vs. untreated control sample by two-way ANOVA and Dunnett multiple comparisons test.

Several studies have demonstrated preferential binding of Siglec-8 to α2,3-sialylated, 6′-sulfated glycan structures, namely 6′-*O*-sulfo-sialyl-Lewis^X^ or 6′*-O*-sulfo-3′-sialyl-LacNAc (36–40). We have also shown that sialidase treatment of eosinophils enhances synthetic polymeric ligand binding (31), suggesting that sialylated cis ligands mask Siglec-8 and prevent binding of ligands in trans. However, these cis ligands have not been further characterized. Because of this, experiments were carried out to assess sialic acid levels at the cell surface on eosinophils at baseline and after treatment with sialidase. Sialic acid is typically found at the non-reducing ends of glycans connected to the rest of the glycan structure via α2,3 or α2,6 glycosidic linkages. Thus, we measured levels of sialic acid at the cell surface in these two different linkages using the lectins *Maackia amurensis* lectin (MAL)-II and *Sambucus nigra* agglutinin (SNA), which bind to α2,3-linked and α2,6-linked sialic acid, respectively. We first confirmed that treatment of human peripheral blood eosinophils with *V. cholerae* sialidase enhanced synthetic Siglec-8 ligand binding (**Figure 1A**). Using the same sialidase, we demonstrated that it dose-dependently diminished α2,3-linked sialic acid levels (**Figure 1B**) but had no detectable effect on levels of α2,6-linked sialic acid (**Figure 1C**), consistent with a previous report of linkage selectivity of *V. cholerae* sialidase at these levels of enzyme activity (41). Together, these data indicate that, similar to identified higher-affinity glycan ligands of Siglec-8, these masking cis ligands bear α2,3-linked sialic acid that is essential for interaction with Siglec-8.

### 3.2 Sialylated cis ligands restrain Siglec-8–induced eosinophil death

**Figure 2:**
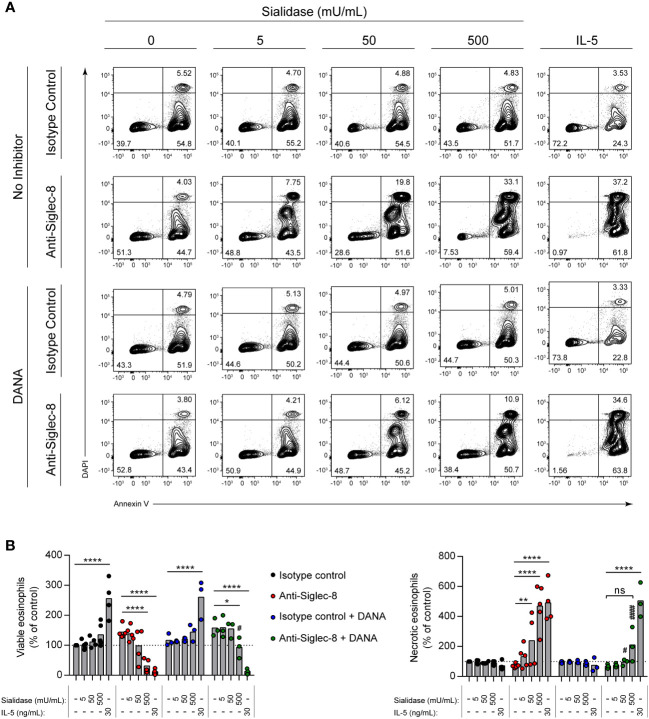
Sialidase treatment of eosinophils licenses Siglec-8 engagement–induced cell death without cytokine priming. Eosinophils were incubated with sialidase at the indicated activities (1 h) or cultured with or without IL-5 (30 ng/mL, 18–24 h) as indicated. Some eosinophils were also pre-incubated with the sialidase inhibitor DANA (250 μM). Eosinophils were then incubated with either anti–Siglec-8 mAb or an isotype control mAb (18–24 h). Cell viability was then assessed by annexin V–DAPI staining by flow cytometry. **(A)** Representative contour plots of annexin V–DAPI viability staining is shown. **(B)** The normalized proportion of viable (annexin V− DAPI−) and necrotic (annexin V+ DAPI+) cells is shown. Each dot represents the results of an independent experiment. Statistical analysis was done by two-way ANOVA with Dunnett’s multiple comparisons test: relative to within-group samples without sialidase treatment or cytokine priming: ns, not statistically significant; *, p<0.05; **, p<0.01; ****, p<0.0001; relative to similarly treated samples in the absence of DANA inhibitor (same antibody and sialidase activity): #, p<0.05; ####, p<0.0001.

Interactions of other Siglecs with sialylated cis ligands can play important functional roles, sequestering the Siglec from targets of inhibitory activity or bringing targets of such activity into close proximity (42). Beyond the ability to limit Siglec-8 interactions with trans ligands, however, the functional impact of the cis ligands of Siglec-8 is unknown. Because the signaling pathway initiated by Siglec-8 antibody engagement is atypical for an ITIM-bearing receptor (11, 35), we hypothesized that Siglec-8 associates in a sialic acid–dependent manner with another receptor that is directly responsible for the observed signaling and cell death. To test this hypothesis, IL-5–primed eosinophils were pre-incubated with sialidase prior to antibody engagement of Siglec-8 and assessment of cell death. In the presence of IL-5 priming, sialidase treatment not only failed to prevent Siglec-8–induced cell death but further promoted it (**Supplementary Figure 1**). Surprisingly, we also discovered that sialidase treatment could overcome the requirement for cytokine priming and license Siglec-8 to cause cell death upon antibody engagement in a dose-dependent manner (**Figures 2A, B**). Eosinophil death following surface sialic acid cleavage and Siglec-8 engagement reached levels similar to those observed in IL-5–primed eosinophils, despite the fact that IL-5 priming did not significantly impact cell-surface sialic acid levels (**Figures 1B, C**). To ensure that this effect was due to the enzymatic activity of the sialidase, the sialidase inhibitor 2-deoxy-2,3-dehydro-*N*-acetylneuraminic acid (DANA) was used. Sialidase inhibition significantly reduced Siglec-8–induced cell death (**Figures 2A, B**), consistent with its ability to impede removal of α2,3-linked sialic acid (**Supplementary Figure 2**).

### 3.3 The sialidase-licensed Siglec-8 cell death pathway in eosinophils resembles that licensed by cytokine priming

One possible explanation for the potentiation of Siglec-8 function by sialidase is that disruption of these cis interactions enables greater antibody engagement of Siglec-8 due to reduction of steric hindrance or greater epitope accessibility via conformational changes of Siglec-8. However, no enhancement of anti–Siglec-8 mAb binding was observed upon sialidase treatment of eosinophils (**Supplementary Figures 3A, B**). In contrast, there was a slight increase in Siglec-8 antibody binding following IL-5 priming, consistent with previously reported results (43).

**Figure 3:**
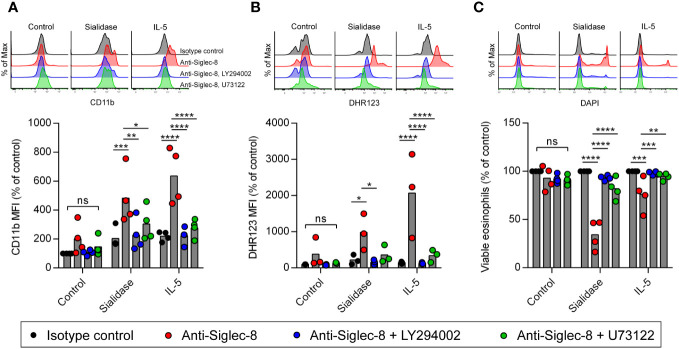
Cell death pathway induced by Siglec-8 engagement following sialidase licensing resembles that observed in cytokine-primed eosinophils. Eosinophils were incubated with sialidase (500 mU/mL, 1 h), IL-5 (30 ng/mL, 18–24 h), in medium without enzyme or cytokine supplementation (Control). Cells were then incubated with the PI3K inhibitor LY294002 (5 μM), the phospholipase C inhibitor U73122 (100 nM), or diluent prior to the addition of anti–Siglec-8 or isotype control mAb. Following 4 h of antibody exposure, levels of cell-surface CD11b **(A)**, intracellular ROS measured by DHR123 MFI **(B)**, and cell viability determined by DAPI exclusion **(C)** were assessed by flow cytometry. Data are representative (top) or normalized to levels observed in untreated samples incubated with isotype control mAb (bottom) and represent the means and individual values of four **(A, C)** or three **(B)** independent experiments. Statistical analysis was done by two-way ANOVA with Dunnett’s multiple comparisons test: ns, not statistically significant; *, p<0.05; **, p<0.01; ***, p<0.001; ****, p<0.0001.

Another possibility is that sialidase treatment of eosinophils facilitates the activation of CD11b/CD18 integrin rather than intrinsic Siglec-8 signaling, as sialylation has been reported to regulate CD11b/CD18 function on neutrophils and microglia (44–46). It is also possible that sialidase-licensed Siglec-8–induced cell death occurs through a distinct pathway that does not mirror the pathway licensed by IL-5 priming. In order to examine these possibilities, we measured cell-surface upregulation of CD11b and production of reactive oxygen species (ROS) and determined the necessity of the signaling molecules PI3K and PLC for these cellular events and cell death after Siglec-8 antibody engagement. No CD11b upregulation or ROS production was observed in isotype control mAb–treated cells following sialidase removal of cell-surface sialic acid (**Figures 3A, B**). Likewise, no significant increases in CD11b expression or ROS were observed in eosinophils upon Siglec-8 antibody engagement without sialidase treatment or IL-5 priming. Both eosinophils that were sialidase-treated and those primed with IL-5 responded to anti–Siglec-8 mAb by augmenting cell-surface CD11b levels and generating ROS. Furthermore, the activities of PI3K and PLC were required for these effects, consistent with our previous findings regarding Siglec-8 signaling (35), although PLC inhibition failed to statistically significantly block ROS production in sialidase-treated eosinophils. Our prior work found that, like PI3K and PLC, Syk was necessary for Siglec-8–induced upregulation of CD11b as well as cell death. Using pharmacologic inhibition of Syk in conjunction with sialidase treatment, we find that Syk is likewise necessary for sialidase-licensed Siglec-8–induced cell death (**Supplementary Figures 4A, B**). Together, these data indicate that the relevant effect of sialidase treatment in this cell death pathway is on Siglec-8 function directly rather than on CD11b/CD18 activation. Finally, and notably, while little cell death is typically observed after just 2 hours of Siglec-8 engagement, sialidase-treated eosinophils were found to have undergone substantial levels of cell death at this time point (**Figure 3C**). Only modest levels of cell death were observed with IL-5 priming at such an early timepoint (**Figure 3C**), indicating that the kinetics of Siglec-8–mediated eosinophil death are more rapid following sialidase treatment.

### 3.4 Sialidase treatment of human MCs facilitates Siglec-8 engagement–induced cell death

**Figure 4:**
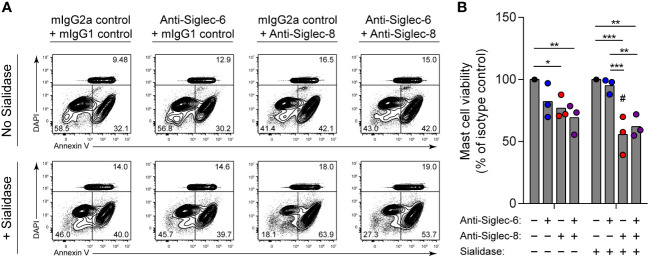
Mast cells are susceptible to Siglec-8 engagement–induced cell death that is potentiated by sialidase treatment. Primary human skin-derived mast cells were pre-incubated with 300 mU/mL *V. cholerae* sialidase for 1 (h) Mast cells were then washed and incubated with anti–Siglec-6 (mouse IgG2a), anti–Siglec-8 (mouse IgG1), and/or their respective isotype control mAbs as indicated for 18–24 h before assessing cell viability by annexin V–DAPI staining and flow cytometry. Data are representative **(A)** or normalized to cell viabilities of the isotype control–treated samples and represent the overall means and individual values of three independent experiments **(B)**. Statistical analysis was done by two-way ANOVA with Tukey’s multiple comparisons test: *, p<0.05; **, p<0.01; ***, p<0.001 relative to indicated within-group samples; or Šídák’s multiple comparisons test: #, p<0.05 relative to the same antibody treatment without sialidase pretreatment.

Engagement of Siglec-8 on human MCs, unlike on eosinophils, has been reported to have no impact on cell viability (15). Instead, Siglec-8 recruits protein tyrosine phosphatases that participate in inhibitory signaling that diminishes MC activation (13–15). The reason for these disparate functions of Siglec-8 in different cell types is unclear. Similarly, antibody ligation of a related receptor, Siglec-6, on MCs inhibits stimulatory signaling and reduces MC activation but does not cause cell death (34, 47). However, Siglec-6 has a binding preference for α2,6-linked sialic acid, at least through its canonical sialoglycan binding pocket (48–50). We therefore hypothesized that removal of α2,3-linked sialic acid from the surface of human skin-derived MCs would sensitize these cells to cell death induced by Siglec-8 but not Siglec-6. Following sialidase treatment, MCs were incubated with antibody against Siglec-8, Siglec-6, or both. Consistent with our hypothesis, Siglec-8–mediated MC death was quite modest at baseline but greatly enhanced following pre-exposure to sialidase (**Figures 4A, B**). In contrast, engagement of Siglec-6 did not result in a significant effect on viability under either condition, and exposure to both anti–Siglec-6 and anti–Siglec-8 antibody resulted in effects like those of Siglec-8 antibody alone. These experiments have uncovered a previously unknown and important contribution of Siglec-8 cis ligands on MCs for constraining Siglec-8–mediated cell death.

## 4 Discussion

Siglec-8 antibody engagement on cytokine (i.e., IL-5, GM-CSF, or IL-33)-primed eosinophils has long been known to induce cell death (9, 10). This pathway is dependent on CD11b/CD18 integrin–mediated adhesion and ROS production by NADPH oxidase and occurs through a signaling pathway involving Syk, PI3K, and PLC, among other signaling molecules (11, 12, 35). However, no apparent effects are observed on eosinophils in response to antibody engagement of Siglec-8 in the absence of cytokine priming or secondary antibody crosslinking, and the necessity for these factors has not been explained. Furthermore, Siglec-8 antibody engagement has not been found to impact MC viability in the presence or absence of SCF or secondary antibody crosslinking (15). We have previously shown that Siglec-8 is partially masked by interactions in cis with sialylated ligands on human eosinophils (31). We demonstrate here that these masking cis ligands bear α2,3-linked sialic acid by using a sialidase that exhibits linkage specificity at the enzyme activities used in this study (41). Given that all sialoside ligands identified for Siglec-8 contain α2,3-linked sialic acid and no α2,6-linked sialic acid–containing glycans have been found to interact with Siglec-8, this result is not surprising (36–40).

Cis interactions of Siglec family members play a number of important roles, such as setting an affinity/avidity threshold for interactions with ligands in trans, sequestering the Siglec from targets of inhibitory signaling, or bringing the Siglec into proximity with a target of inhibition (23, 42). We show here that Siglec-8 interactions with cis sialylated ligands restrain cell death induction and that the need for cytokine priming can be overcome by enzymatically removing α2,3-linked sialic acids from the cell surface. The pathway initiated by Siglec-8 engagement in sialidase-treated eosinophils mimics that characterized in IL-5–primed eosinophils, including a requirement for Syk activity and the cell-surface upregulation of CD11b and the generation of ROS, both of which are dependent on PI3K and PLC activities (11, 12, 35). This raises the possibility that cytokine priming of eosinophils achieves its effects on Siglec-8 function by disrupting these cis interactions. Although no generalized effects of cytokine priming on the cell-surface sialic acid profile are seen, it remains possible that cytokine priming disrupts Siglec-8 cis interactions through other means, such as the conformational alteration of the specific glycoconjugates that engage Siglec-8 or their movement to a different membrane microdomain such that the glycans remain accessible to MAL-II but inaccessible to Siglec-8. Alternatively, priming may cause the endocytosis of the Siglec-8 cis ligands that may represent a minor component of the pool of cell-surface glycans recognized by MAL-II. Indeed, the identities of the cell-surface glycoconjugates that interact with Siglec-8 are currently unknown, and further study will be needed to characterize them. While the global removal of cell-surface α2,3-linked sialic acid is effective, it may be beneficial to target the relevant glycoconjugates that bind to Siglec-8 to deplete eosinophils and MCs. Our results also indicate that direct disruption of cis ligand interactions may accelerate Siglec-8–induced cell death.

Previous reports have indicated that cell-surface sialylation regulates CD11b/CD18 integrin function on neutrophils and microglia. Feng et al. demonstrated that human neutrophils mobilize endogenous sialidase to the cell surface upon activation and that desialylation of either neutrophils or the CD11b/CD18 ligand ICAM-1 enhanced binding and adhesion (46). A subsequent study found that human neutrophils remove α2,3-linked (but not α2,6-linked) sialic acid from their cell surface during transepithelial migration (TEpM) and that sialidase inhibition reduced TEpM as well as fMLF-induced CD11b conformational activation, degranulation, ROS production, and signaling (45). Likewise, activation of murine microglia leads to enhanced cell-surface sialidase activity, and desialylation of microglia using sialidase or a sialyltransferase inhibitor increases phagocytosis and neuronal loss in co-culture in a CD11b-dependent manner (44). The precise mechanisms underlying these effects of sialylation on CD11b/CD18 integrin have not been elucidated, but it is notable that these cells express Siglecs that, similar to Siglec-8, recognize α2,3-linked sialylated ligands, including Siglec-9 and its murine ortholog, Siglec-E (reviewed in (51)) that may restrain integrin activity. Indeed, mice lacking functional Siglec-E reveal a role for Siglec-E in the regulation of CD11b/CD18 integrin–mediated function in mouse neutrophils, although the effects are complex, with apparent roles for Siglec-E in dampening CD11b-dependent neutrophil recruitment and Syk and p38 phosphorylation but promoting CD11b-dependent ROS production and Akt phosphorylation (52, 53). Impaired ROS production was observed in functionally Siglec-E–deficient neutrophils in response to either LPS administration or fibrinogen (53). In our study, no endogenous sialidase activity was apparent, there were no global alterations of the cell-surface sialic acid profile in response to IL-5 priming, and the sialidase inhibitor DANA exerted no effect in the absence of exogenous sialidase, suggesting that eosinophils may alter cell-surface sialylation or sialoside ligands via a distinct pathway from those employed by neutrophils and microglia. Importantly, there was also no effect of sialidase treatment on CD11b upregulation, ROS production, or cell death in the absence of Siglec-8 ligation, and downstream effects of engagement were mediated by signaling similar to the pathway we have previously described for Siglec-8 engagement in IL-5–primed eosinophils (35), indicating that de-sialylation does not promote CD11b/CD18 integrin function directly in this system.

The consequences of antibody engagement of Siglec-8 on primed eosinophils and MCs are disparate, despite the fact that there are no known changes in the structure of the receptor between these two cell types. These distinct outcomes could be explained by differences in the sets of downstream signaling molecules or in the propensity of these cell types to produce ROS or engage a particular cell death pathway. An alternative explanation, supported by this work, is that cis interactions govern the functional consequences of Siglec-8 engagement. We show that antibody ligation of Siglec-8 on primary human MCs produces a low level of cell death at baseline but that antibody engagement following sialidase treatment reduces MC viability by about half within 24 hours. This result is consistent with the relatively high abundance of sialic acid on the surface of human MCs compared to human eosinophils (54), which suggests that, while Siglec-8 is capable of inducing cell death in both cell types, characteristic outcomes are dictated by cell surface glycosylation. Further study will be necessary to determine whether Siglec-8 on MCs utilizes a similar cell death pathway to that characterized in eosinophils and whether this pathway is engaged under physiological conditions. It is interesting that Siglec-8 engagement–induced cell death requires a preliminary step, whether it be cytokine priming or sialic acid removal, that appears to release Siglec-8 from a constrained state. Given that cytokine priming is the more physiologic signal for this *in vivo*, it is logical to hypothesize that the natural role of Siglec-8 is to clear activated eosinophils, and perhaps MCs, during the resolution of type 2 inflammation. However, the physiologic signals that license Siglec-8 to achieve this effect in MCs, if any, have not been identified. Nevertheless, whether this pathways is engaged naturally to clear MCs or not, it could be exploited therapeutically to deplete MCs in disease states.

Currently, while there are several therapeutic options to block the activities of mediators released from MCs, there are few options to more globally address MC-mediated inflammation. Siglecs and other inhibitory receptors present on MCs are good therapeutic targets to inhibit MC activation more generally. Indeed, both Siglec-8 and Siglec-6 inhibit MC activation through a variety of different pathways to some degree (14, 34). However, antibody targeting of Siglec-8 is unable to deplete MCs by antibody-dependent cellular cytotoxicity in the same way as eosinophils, presumably due to a lack of cytotoxic NK cells in tissues in which MCs are found (55, 56). MC depletion strategies have largely focused on KIT, but small-molecule inhibitors of KIT often have substantial off-target effects and neither small-molecule inhibitors nor biologics are capable of distinguishing MCs from other KIT+ cells types, including hematopoietic stem cells, megakaryocytes, spermatogonia, primed CD8+ T cells, interstitial cells of Cajal, melanocytes, and T1R3+ taste cells (57–63), raising concerns about potentially deleterious on-target effects. Our results demonstrate that rapid MC depletion can be achieved *in vitro* by ligating Siglec-8 after removing cell-surface sialic acid. Because Siglec-8 is selectively expressed on eosinophils, MCs, and at low levels on basophils (8), this approach offers promise as a means to selectively and effectively deplete MCs in patients with MC-mediated diseases.

## Data availability statement

The data presented in the study are deposited in the ImmPort data repository, accession number SDY2365.

## Ethics statement

The studies involving humans were approved by the Institutional Review Board of Northwestern University Feinberg School of Medicine. The studies were conducted in accordance with the local legislation and institutional requirements. The participants provided their written informed consent to participate in this study.

## Author contributions

YC: Investigation, Writing – review & editing. CR: Investigation, Writing – review & editing. BB: Conceptualization, Funding acquisition, Supervision, Writing – review & editing. JO: Conceptualization, Formal Analysis, Funding acquisition, Investigation, Supervision, Writing – original draft.
