# Supplementary material for: Interactions between Siglec-8 and endogenous sialylated cis ligands restrain cell death induction in human eosinophils and mast cells
Source: Front Immunol. 2023 Oct 20;14:1283370. doi: 10.3389/fimmu.2023.1283370 (PMC10623328; doi:10.3389/fimmu.2023.1283370)
Supplement: Supplementary file 1 [file DataSheet_1.pdf]

Supplementary Material

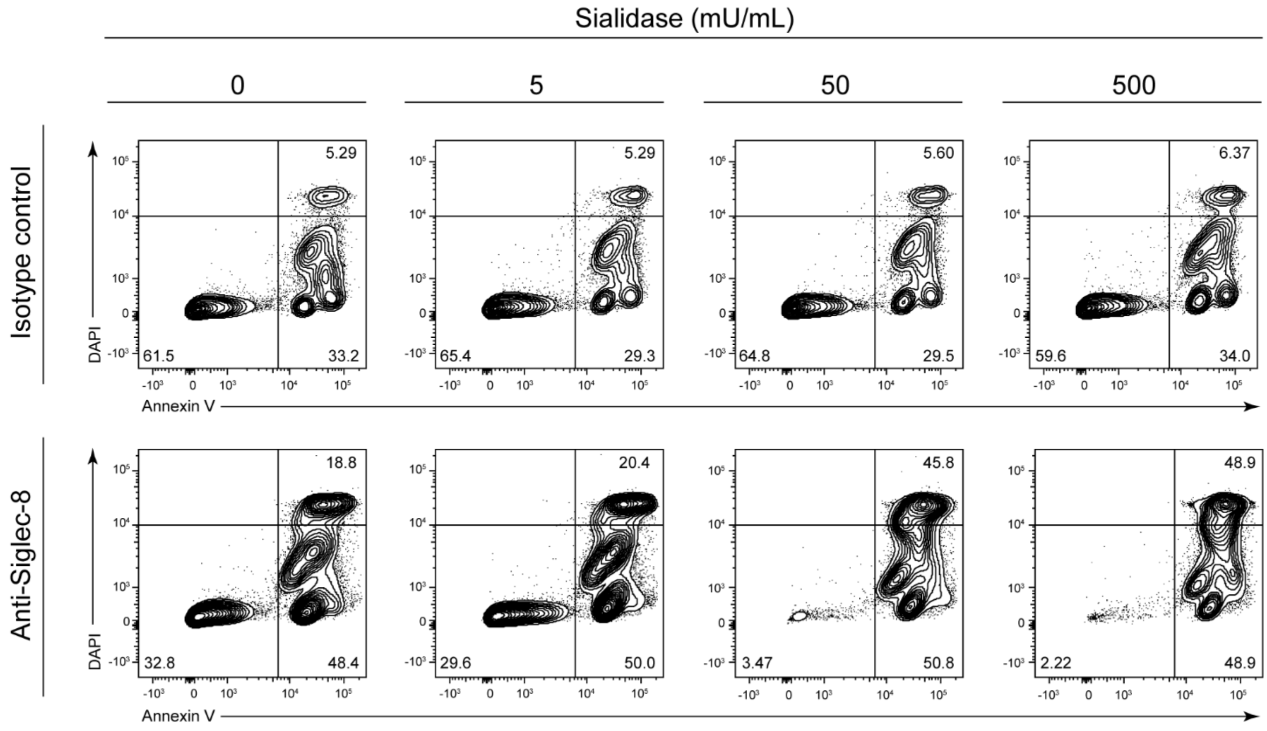

Supplementary Figure 1. Sialidase removal of cell-surface sialic acid promotes Siglec-8-induced cell death in IL-5-primed eosinophils. Eosinophils were incubated with IL-5 at 30 ng/mL for 18-24 h prior to the addition of *V. cholerae* sialidase at the indicated activities. After 1 h, anti-Siglec-8 mAb or mIgG1 control mAb was added at 2.5 µg/mL. Eosinophils were stained with fluorophore-conjugated annexin V and DAPI after 18-24 h, and cell viability was assessed by flow cytometry. Results are representative of two independent experiments.

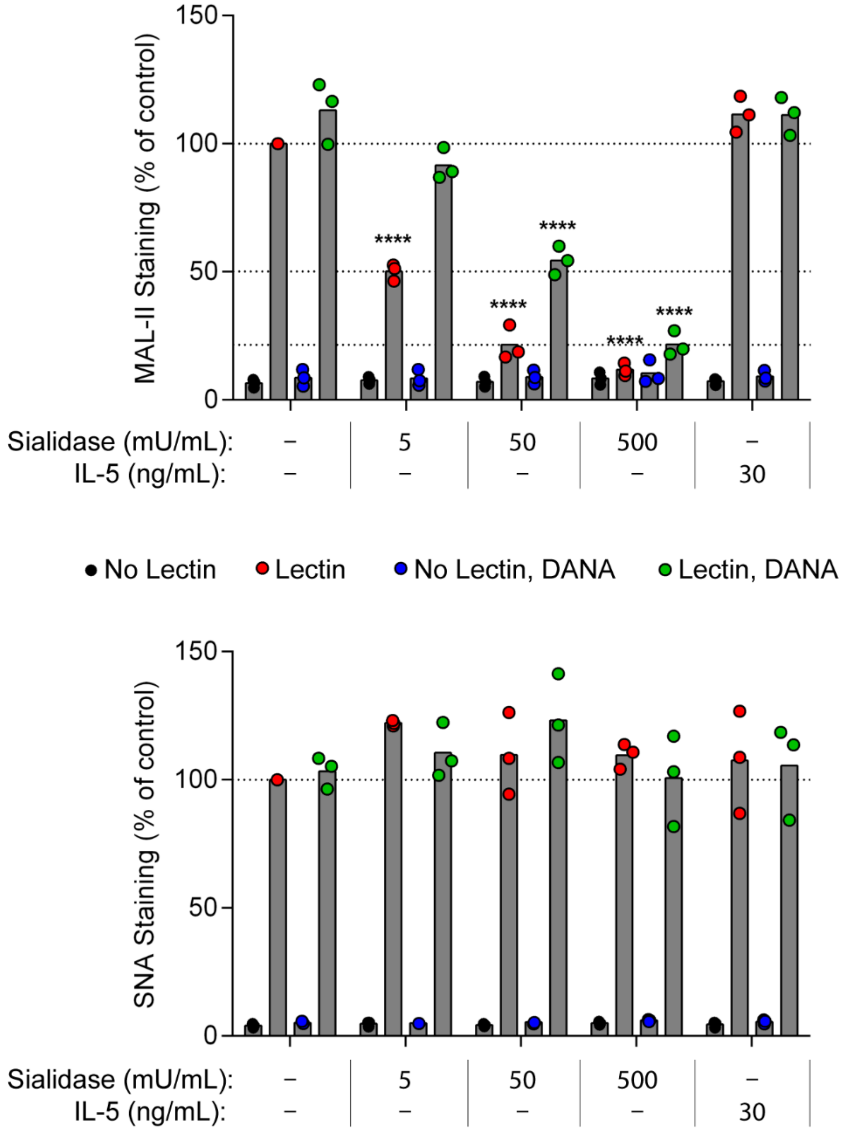

Supplementary Figure 2. Sialidase enzymatic activity is reduced but not completely eliminated by the sialidase inhibitor DANA. Eosinophils were incubated with sialidase at the indicated activities (1 h), primed with IL-5 (30 ng/ml, 18-24 h), or left untreated as indicated, with or without 1-h pre-incubation with the sialidase inhibitor DANA (2,3-dehydro-2-deoxy-N-acetylneuraminic acid). Eosinophils were then incubated with biotinylated MAL-II or SNA lectin to detect cell-surface α2,3- or α2,6-linked sialic acid, respectively. Surface-bound lectin was detected with fluorophore-conjugated streptavidin by flow cytometry. Lectin binding was quantified by normalizing gMFI to that of untreated control eosinophils. Dotted lines indicate baseline MAL-II and SNA binding as well as MAL-II binding at each sialidase activity level in the absence of DANA. Statistical analysis was done by two-way ANOVA with Dunnett's multiple comparisons test: ****, $p<0.0001$ relative to lectin-stained untreated control samples. Each dot represents the results of an independent experiment.

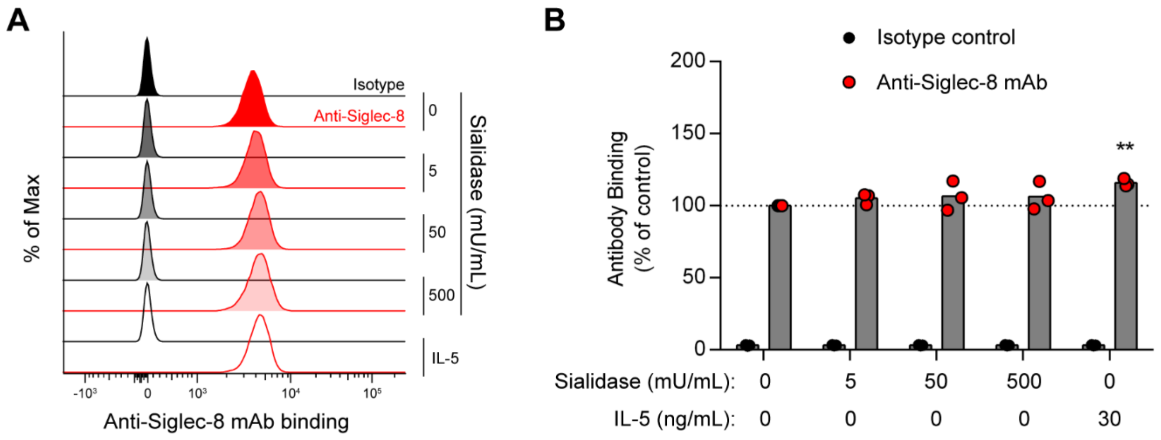

Supplementary Figure 3. Sialidase treatment has no impact on anti-Siglec-8 mAb binding. Eosinophils were incubated with sialidase at the indicated activities (1 h) or cultured with or without IL-5 (30 ng/ml, 18-24 h) as indicated. Binding of fluorophore-labeled anti-Siglec-8 mAb or isotype control mAb was assessed by flow cytometry (**A**) and quantified by normalizing MFI of anti-Siglec-8 mAb binding to that of eosinophils without enzyme or cytokine treatment (**B**). Statistical analysis was done by two-way ANOVA with Tukey's multiple comparisons test: **, $p<0.01$ relative to untreated control samples. Data are representative (**A**) or indicate the means and individual values from 3 independent experiments (**B**).

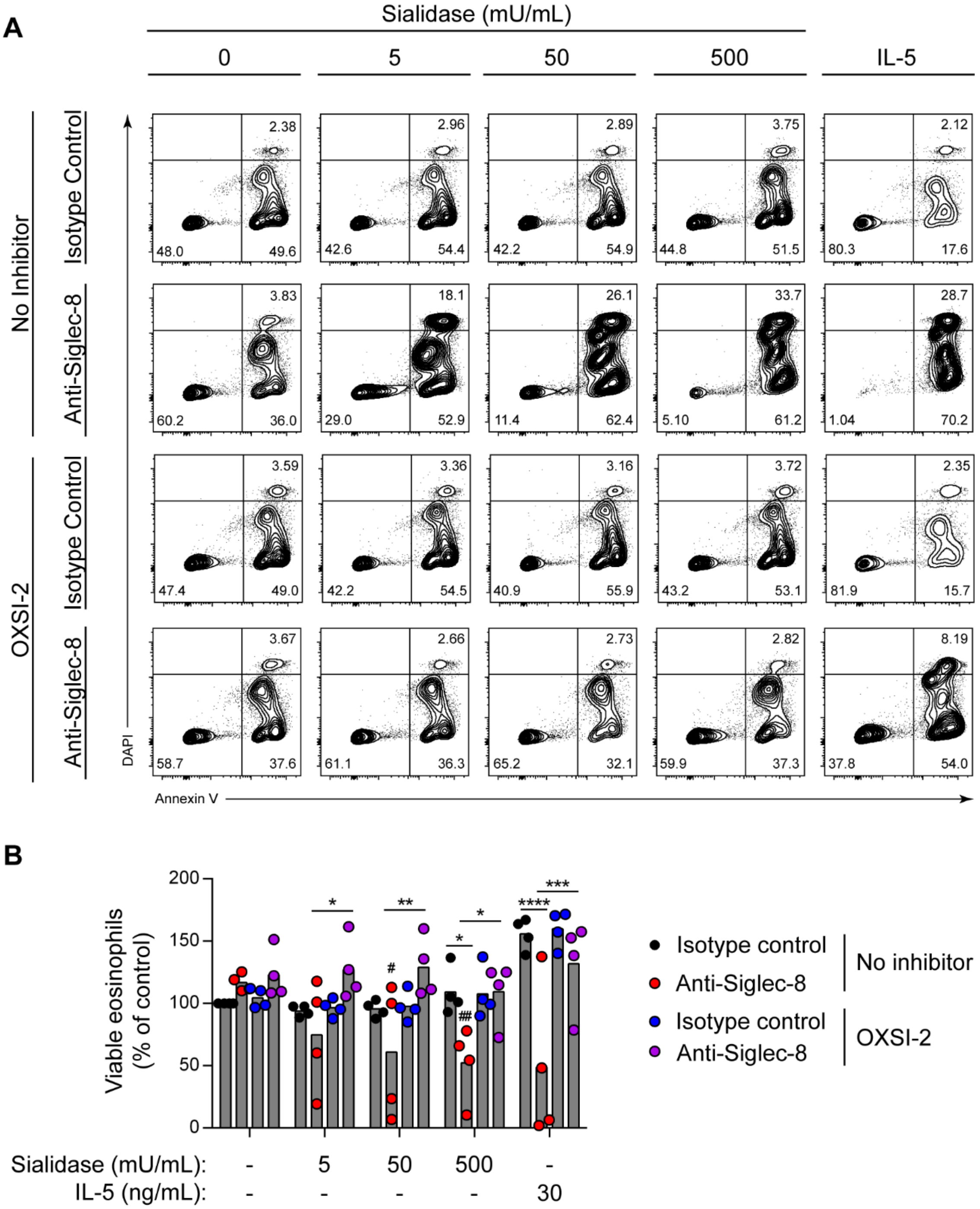

Supplementary Figure 4. Syk activity is required for Siglec-8 engagement-induced cell death licensed by sialidase. Eosinophils were cultured with or without IL-5 (30 ng/ml, 18-24 h) and incubated with sialidase (1 h) as indicated. In addition, eosinophils were pre-incubated with the Syk inhibitor OXSI-2 at 667 nM for 30 min before the addition of anti-Siglec-8 mAb or mouse IgG1 isotype control mAb. After 18-24 h, the eosinophils were stained with fluorophore-conjugated annexin V and DAPI and analyzed by flow cytometry. Cell viability was normalized to that of

eosinophils incubated with the isotype control mAb in the absence of enzyme or cytokine. Data are shown as representative flow plots (**A**) and as normalized cell viabilities (**B**). Statistical analysis was done by two-way ANOVA with multiple comparisons tests as appropriate: Within-group comparisons: *, $p<0.05$; **, $p<0.01$; ***, $p<0.001$; ****, $p<0.0001$ calculated by Tukey multiple comparisons test. Inter-group comparisons: #, $p<0.05$; ##, $p<0.01$ vs. the same antibody-inhibitor treatment in the no-sialidase group. Data are representative (**A**) or indicate the means and individual values from 4 independent experiments (**B**).
